# 

**Published:** 1915-10-16

**Authors:** 


### CONTENTS.

PAGE

**The Training of Memory** ... ... ... ... 41
**Another Raid on the Civil Hospitals** ... ... 42
**Hospital and Institutional News** ... ... ... 43
Our Special War Number—A Notable Absence of Apprehension—The "Star and Garter" to Disappear—An Excellent Choice—The New Huddersfield War Hospital—Invaluable Personal Service—The Cost of Panic or Worse—A New Work for the Institute of Secretaries: War Comforts—An Official Badge—Indian Prisoners in Germany—A Superintendent's House White Elephant — Injudicious Publicity — Expensive Economy at Preston—The Distribution of Infectious Diseases—The Coming Value of Notification Statistics — Visitors to the Wounded—No New Superintendent at the Park Hospital—War Profits and Hospital Contributions—Emergency Treatment of Electrical Shock—An Interesting Piece of History Recalled—Uniformity in First-Aid Methods—The "Sweated Industry" of Tuberculosis Work—A Countermarch Against "Twilight Sleep"—Camberwell's Premium on Personal Filth—This Week's Drug Market.
**Tetanus in War** ... ... ... ... ... ... 49
Value of the Serum Treatment.
**Pubiotomy, Cæsarean Section, and Induction** 50
**Some General Effects of War** ... ... ... 51
I.—Upon the Voluntary Hospitals.

PAGE

**Hospital Barges for the Wounded** ... ... 52
**The Editor's Letter-Box** ... ... ... ... 53
The Vacant Secretaryship at Norwich: Hundreds of Applications or None?
Hospital Saturday, 1915.
**Round the World** ... ... ... ... ... 54
Fellow-Workers and the Roll of Honour.
**The Heating of Hospitals** ... ... ... ... 55
IV.—The Manchester Royal Infirmary.
**War Equipment** ... ... ... ... ... ... 56
**The Regimental Stretcher-Bearers** ... ... 57
Their Heroism and Grit.
**Hospital Economy in War-Time** ... ... ... 57
**Passing Topics of the Day** ... ... ... ... 58
Red Cross Sisters Murdered by Germans—Are Telephone Mouthpieces Harmless?—Wounded Needs in Alexandria.
**The Norfolk and Norwich Hospital** ... ... 58
**The "Massage Establishments" Scandal** ... 59
Lords' Select Committee's Inquiry and Decision.
**A Glance at Some Contemporaries** ... ... 60
The Builders Again at Guy's—Deceptive Disinfectants—Fire Protection in Old Hospitals—The Abuse of Drugs—The Hygiene of Illumination—Refusal to Submit to Operations.
**Personalia** ... ... ... ... ... ... iv
**The Institutional Worker** ... ... (Suppl.) 1

